# Environment, Environmental Crimes, Environmental Forensic Medicine, Environmental Risk Management and Environmental Criminology

**DOI:** 10.3390/healthcare10020263

**Published:** 2022-01-29

**Authors:** Michelangelo Bruno Casali, Guido Vittorio Travaini, Carlotta Virginia Di Francesco, Umberto Rosario Genovese

**Affiliations:** 1 Institute of Legal Medicine, University of Milan, 20122 Milan, Italy; carlotta.difrancesco@unimi.it (C.V.D.F.); umberto.genovese@unimi.it (U.R.G.); 2 Department of Oncology and OncoEmatology, University of Milan, 20122 Milan, Italy; 3 MELTECNAM Lab (Laboratory of TechnoEnvironmental Forensic Medicine), University of Milan, 20122 Milan, Italy; 4 School of Medicine, Vita & Salute San Raffaele University, 20132 Milan, Italy; travaini.guido@unisr.it

**Keywords:** forensic medicine, environment, virtual environment, green crime, green criminology, environmental justice, environmental forensic medicine (EFM), Artificial Intelligence (AI), environmental pollution

## Abstract

Forensic medicine has always held the human environment, either seen as a source for pathological agents or the background of judicial events, in great consideration. The concept of the environment has evolved through time, expanding itself to include all the physical and virtual sub-spaces in which we exist. We can nowadays talk of technoenvironmental reality; virtual spaces exploded because of the COVID-19 pandemic making us come to terms with the fact that those are the places where we work, where we socialize and, even, where we meet our doctors and can be cured. Artificial Intelligence (AI) has contributed to shaping new virtual realities that have got their own rules yet to be discovered, carved and respected. We already fight a daily battle to save our natural environment: along with the danger of green crimes, comes the need for environmental justice and environmental forensic medicine that will probably develop a forensic branch and an experimental branch, to implement our technical culture leading to definition of the real dimension of the risk itself to improve the role of legal medicine in the Environmental Risk Management. While green criminology addresses widespread green crimes, a virtual environment criminology will also develop, maybe with a contribution of AI in the justice field. For a sustainable life, the environmental revolution must rapidly take place, and there is the need for a new justice, a new forensic medicine and a new criminology too.

## 1. Introduction

Forensic medicine has always been strictly focused on human environment, traditionally intended as either the possible source for some pathological agents or the interacting background for some judicial events [1].

In the XXI Century, a new description of the concept *environment* is probably needed, and this new kind of human environment can be maybe defined as the cumulative space for all the human actions and all the human interactions: a comprehensive space where physical sub-spaces and virtual sub-spaces do routinely coexist. As the nature first created the *physical environment*, the human technology has then created the *virtual environment.*

The XXI Century reality is, therefore, *technoenvironmental*, as long as its common living spaces are both physical and virtual. The clinical medicine too is changing its view about the environment: through an OMICS approach, the clinical medicine is now looking at the Envirome, intended as the dynamic combination among natural environment, social environment and personal environment [2].

Everyday millions of human beings unconsciously cross—one by one—many different physical and virtual spaces: any of these interactions can be relevant for justice and, therefore, for either forensic medicine and criminology.

The Internet was maybe the very first virtual space ever [3], and its evolution has now come to a point where such a space is alternatively a work-space, a game-space, a social-space and, finally, a crime-space too [4]. Virtual spaces have been also extremely dilated because of the COVID-19 pandemic for social, medical, and work purposes [5]. In the meantime, new frontiers and new shapes for the virtual environment have been produced by the Artificial Intelligence (AI), with its basic scheme made up by an original input, a more or less indefinite powerful computation and a final output [6]. Talking about AI, the virtual space often looks such as a *black box* [7] with the consequent deep issue about the limits of the human comprehension of AI.

AI-based virtual environment is mainly a very fast and very compressed input-output reality with a huge intermediate sacrifice for the human understanding from the outside. On the other hand, the physical environment is a gradual reality featuring factual chains made up by many different visible or understandable intermediate steps. Physical and virtual environments are then very different, the former being traditional to humans and the latter being new and unknown. 

As it is clear mainly from the AI revolution, the virtual environment has got its own rules yet to be discovered, carved and respected.

The XXI Century technoenvironmental world will use both old physical rules and new virtual rules, but some of the old physical rules must be probably refreshed or reinvented: the XXI Century must therefore support a new technical approach to the natural environment too [8]. Rules-laws-crimes is an obvious trilogy indeed. 

As human beings, we all fight a daily battle to save our natural environment [9,10]. Plastic pollution, poisonous gases, chemical residues, noise pollution and wild deforestations are good examples of collective enemies to be immediately fought [11,12,13,14]. Such a fight (just like every other human action) has got, obviously, a judicial face too, the face of the so-called *green crimes*. In its simplest version, green crimes are the face of unrespected environmental rules.

Existing therefore green crimes, our society has a strong need for an effective *environmental justice* (EJ) and an effective *environmental forensic medicine* (EFM). 

## 2. Environmental Justice, Environmental Forensic Medicine and Environmental Risk Management

An effective EJ basically aims at punishing green crimes and at compensating for damages produced by green crimes. 

The EFM will have probably a double-faced identity: it will be at the same time a pure forensic branch (serving the justice) and, also, a very prolific experimental branch (serving the science). As in a coin, these two faces are firmly bound to each other.

### 2.1. EFM as a Pure Forensic Branch

As a pure forensic branch, the EFM will deal with individual and collective consequences of green crimes. 

Individual consequences are well represented by harms and physical injuries occurred to a small number of some exposed people (for example, asbestos-related diseases in asbestos-exposed people).

On the other hand, collective consequences can have the proper shape of multiple and widespread harms (maybe judicially treated in a class action frame) or the alternative shape of a widespread risk factor. A pure risk factor is something not come to in the final stage of a causal factor. 

Green crimes can be, therefore, divided into two different groups: *event crimes* versus *risk crimes*.

Event crimes will stimulate the EFM in a quite traditional way (causal factors and individual causation as the final technical focuses), while risk crimes will stimulate the EFM in a quite new way (risk factors and general causation as the final technical focuses).

Green event crimes usually bear the cross of a very difficult causal diagnosis about the relationship between the environmental risk factor and the alleged connected pathology. Risk crimes do have a more epidemiological dimension than event crimes and the connected medical investigation will be probably more focused on causal plausibility than on a strict individual causality.

About risk crimes, a new task for EFM will be probably the search for the people at risk and the definition of the real dimension of the risk itself: such a new epidemiological role for the forensic medicine will probably have important repercussions on primary and secondary prevention of environmental pathologies. It is the same path already seen from the traditional legal medicine applied to the Medical Malpractice (crime punishment, damage compensation) to the Clinical Risk Management (prevention of medical mistakes): the issue is now about the role for a renewed legal medicine in the Environmental Risk Management (ERM) [15,16].

As all other human risks, the environmental risk—here defined as the probability of an adverse effect on humans and/or on environment resulting from a given exposure to toxic substances [17]—must be investigated, recognized, managed and communicated [18]. Together with clinical toxicology, EFM can help investigate the environmental risk adding biological exposure maps to the preliminary group of contamination maps, exposure maps and hazard maps: the result would be the production of very powerful synoptic risk maps as effective tools for pathological prevention [19].

As detailed below, forensic contributions to environmental science and ERM could obtain the shape of maps of biological exposure coming from experimental works performed on non-selected necroscopic samples [20].

EJ will probably use such forensic contributions—combined with geographical maps—as a primary tool in investigating and punishing environmental crimes. Talking about environmental risk and dealing with risk crimes, EFM will inevitably have a strong bibliographic feature, where scientific coverage laws will really make the difference [21]: the focus is the population at risk and the risk—other than the individual case—can be well described by cohort studies. It finally sounds, once again, as if the fight in the courts between the good science and the junk science [22,23], a hot topic already for the traditional forensic medicine [24]: a lot of work for EFM will be made to investigate the quality of available coverage laws [25] and selecting only the best covering scientific evidence will be crucial for EFM and for EJ. Moreover, many coverage laws are yet to be created. Once again, AI will help this way by making a strong methodological and bibliographic revolution [26].

### 2.2. EFM as an Experimental Branch

Both EJ and clinical medicine need good scientific evidence.

To create scientific laws about environmental risk and environmental harms, there is the crucial need for a deeper scientific knowledge about the biological effects of environmental exposures and the experimental EFM must therefore cooperate with the clinical toxicology and the clinical environmental medicine to implement such a technical culture. Wide unselected autoptic populations can serve as unique sources for tissue samples to be investigated [27]: such works can provide data about the population reference values for external toxics (values from healthy people died of a violent death), that are the terms then to compare with the singular values from the specific case alleged for an environmental crime. In the paradigmatic case of asbestos exposure, the Helsinki Consensus Conference and its updates give a clear description of both the clinical and the forensic meaning of the reference values [28]. Outside the goals of EJ, the reference values are also very important for biomonitoring and for performing all the stages of prevention of environmental and occupational diseases [29,30,31,32,33]. Together with data coming from clinical works [34,35,36], only forensic necroscopic data can lead to a better understanding of the pathophysiology of lethal and sublethal environmental poisonings. When parenchymal reference values are needed, the forensic necroscopic sample is better than the clinical bioptic sample for two different reasons: the former comes from healthy people, and it allows a wide parenchymal mapping.

A deep and complete environmental culture (also coming from EFM experimental works) is absolutely needed to create good regulatory measures and laws and good laws will help protect ourselves and our planet.

Only a mature double-faced EFM will then address all the increasing issues of EJ and ERM.

## 3. Environmental (Green) Criminology

Together with the EFM, the Green Criminology has the task of analyzing all the criminal processes connected to the natural environment [37,38,39]. The green criminology addresses forms of crime that harm the environment, but that are often ignored in the mainstream criminological research [40]. Green crimes research does already exist [41,42], but its importance will predictably rise up in the next times. Green crimes cause both direct and indirect forms of harm, the former of which affects the ecosystem while the latter comes as a consequence of direct harms. Compared to typical criminal harms, green crimes are much more widespread [43]. Policies intended to control crime and address biases in law must incorporate green criminology in order to reduce the environmental pollution. The green criminology analyzes the variety of green victims, including direct ecological harms and indirect harms to human and non-human species [44].

It also discusses the primary kinds of direct environmental harms such as air, land, water, mining and timber crimes, as well as the issue of green policies with respect to efforts to reduce environmental pollution.

Beside the green criminology focused on the physical environment, a *virtual environment criminology* will certainly develop. Environmental Criminology (EC) is a much wider matter than just the green criminology indeed (EC = green criminology + virtual spaces criminology). Internet crimes already lead the way, but new pathways will be probably created by the Artificial Intelligence. After all an AI-based justice has been already theorized [45,46,47].

## 4. Conclusions

According to an up-to-date definition, the human environment is the cumulative space for all the daily human actions and interactions. Natural environments and virtual environments do synergically coexist in a technoenvironmental reality. Nowadays a very new global approach to human environment is then widely required: the main goals are the protection of the natural environment and the regulation of the virtual environment. Both physical and virtual spaces must be better studied and understood: functional rules will come only from good and deep understanding and functional rules will be the effective tools for the prevention of both environmental disease and environmental crimes. In such a perspective, the chain starting from EJ, passing through EFM and EC and, finally, getting to ERM will work as a whole. For a sustainable life, the environmental revolution must rapidly take place: such a revolution does need a new justice (EJ), a new forensic medicine (EFM), an effective environmental risk management (ERM) and a new criminology (EC) too. No doubt that the clinical environmental medicine is ready [48,49]. Is the forensic medicine ready as well?
